# Cascading Effects of Plant Hormone-induced Trait Shifts in *Alnus rubra* on Aquatic and Terrestrial Ecosystem Function

**DOI:** 10.1007/s10886-025-01644-9

**Published:** 2025-09-13

**Authors:** Taryn Y. Broe, Alexia Fabiani, Mirte C. M. Kuijpers, Sara L. Jackrel

**Affiliations:** School of Biological Sciences, Department of Ecology, Behavior & Evolution, University of California San Diego, 9500 Gilman Drive #0116, La Jolla, CA 92093-0116 USA

**Keywords:** Plant defensive chemistry, Litter mass loss, *Alnus rubra*, Riparian ecosystems, Ellagitannins, Diarylheptanoids

## Abstract

While intraspecific trait variation can regulate ecosystem functions, there is limited understanding of whether this variation caused by inducible phenotypic shifts, as opposed to genetic hardwiring, can have such effects. Leveraging the complex inducible defense systems of plants, we found that treating young *Alnus rubra* trees with the plant hormones methyl jasmonate and salicylic acid, which are involved in plant defensive responses to herbivory and/or pathogen stress, triggered shifts in the composition of leaf secondary metabolites and nutrient reallocation among plant tissues. Induction of the jasmonic acid pathway triggered substantial changes among the dosage-dependent ellagitannins and a decline in leaf nitrogen, while induction of the salicylic acid pathway triggered greater restructuring among the cytotoxic diarylheptanoids. Concurrent induction of both pathways caused distinct changes in leaf composition compared to either pathway alone. We also found opposing seasonal trends of these two major classes of secondary metabolites with seasonal declines among diarylheptanoids and increases among ellagitannins. Further, these plant hormone-induced compositional changes in leaves had cascading effects on ecosystems, with litter mass loss rates in both aquatic and terrestrial ecosystems inhibited most by induction of the jasmonic acid pathway, least by induction of the salicylic acid pathway, and effected at intermediate levels by the combined induction of both pathways. These results demonstrate that inducible plant defenses that are mounted against terrestrial stressors can alter rates of litter mass loss in aquatic and terrestrial ecosystems.

**Supplementary Information:**

The online version contains supplementary material available at 10.1007/s10886-025-01644-9.

## Introduction

Biodiversity at the intraspecific scale has been found to rival the effects of among-species diversity in regulating ecosystem functions and community structure (Bassar et al. 2010; Crutsinger et al. 2006; Des Roches et al. 2018). Both shifts in genetic composition and inducible phenotypic variation are elements of intraspecific variation that can be essential for population survival under fluctuating environmental conditions (Agrawal 1998; Barbour et al. 2009; Hendry 2016; Hersch-Green et al. 2011; Miner et al. 2005). While studies have demonstrated that genotypic variation can have broad ecosystem-level effects (Barbour et al. 2009; Crutsinger et al. 2006; Hersch-Green et al. 2011; LeRoy et al. 2006; Whitham et al. 2006), few studies have evaluated the potential implications of inducible trait shifts on whole communities and ecosystems (LeRoy et al. 2020). Therefore, we aimed to evaluate the extent to which inducible phenotypic variation can regulate ecosystem function by testing effects of inducible trait shifts in plants both within and across ecosystem boundaries. We test the effects of inducible phenotypic variation on leaf litter mass loss, which is a proxy for the important ecosystem function of decomposition. We measure litter mass loss in both aquatic and terrestrial ecosystems, which both rely on leaf influx as an important energy source at the base of their respective food webs (Cebrian and Lartigue 2004). To determine whether these induced plant responses have measurable effects in diverse, natural ecosystems, we use a naturally occurring population of genetically variable trees of *Alnus rubra*. This species commonly dominates riparian forests in the Pacific Northwest of North America, while the genus *Alnus* is an important pioneer species throughout riparian forests and forest edges of the Northern Hemisphere (Heuvel 2011; Luken and Fonda 1983; Tarrant and Trappe 2014).

Plants are an ideal model system to evaluate the ecosystem-level effects of inducible phenotypic variation because they have complex defense systems to protect against diverse pathogens and herbivores. These systems are comprised of both a persistent baseline of constitutive defenses, as well as an inducible defense system that consists of two separate but interacting pathways: the salicylic-acid (SA) pathway and the jasmonic-acid (JA) pathway. The SA pathway defends against biotrophic pathogens including fungi and viruses that feed on living plant tissues, while the JA pathway defends against chewing and mining herbivores and necrotrophic pathogens that feed on dead plant tissue (Kunkel and Brooks 2002; Raskin 1992; Wittstock and Gershenzon 2002; Moreira et al. 2018). Additionally, both the SA and JA pathways have been implicated in deterring phloem feeding insects (Thaler et al. 2012). Plant defense responses are further complicated by several additional plant hormones, such as ethylene, abscisic acid and gibberellic acid, that are also involved in mediating inducible defense responses (Thaler et al. 2012; Ma and Ma 2016). Cross-talk between the JA and SA pathways has been documented in model plant taxa, including *Arabidopsis thaliana*, *Solanum lycopersicum* and *Nicotiana* spp., as reciprocal antagonism in that induction of the SA pathway might hinder a plant’s defensive response via the JA pathway, and vice versa (Thaler et al. 2012). However, evidence for reciprocal antagonism has not been universal. Studies have also found asymmetrical effects between the two pathways, as well as synergistic and neutral interactions, with plant responses often highly contingent on the biology of the specific attacker (Moreira et al. 2018). For example, a recent meta-analysis found that induction of the JA pathway by herbivores tended to negatively affect a wide range of subsequent attackers, including those that would induce the SA pathway. In contrast, induction of the SA pathway tended to have weaker and more variable plant-mediated effects for subsequent attackers (Moreira et al. 2018).

Plant inducible defenses that are mounted against herbivore and pathogen stress can include a range of mechanical and chemical responses. Mechanical defenses, such as increased trichome density or thickening of leaves, may deter chewing insects and mammals (Baur et al. 1991; Coley 1983). Chemical defenses, such as tannins and glucosinolates, may be distasteful or toxic to these same consumers as well as to galling insects (LeRoy et al. 2020; Mithöfer and Boland 2012). Dosage-dependent or quantitative chemical defenses, such as tannins, often interfere with herbivore nutrient acquisition and generate oxidative stress when present at relatively high concentrations. In contrast, qualitative chemical defenses, such as glucosinolates, tend to be toxic at low concentrations (Feeny 1976; Stamp 2003; Barbenhenn et al. 2009; Smilanich et al. 2016). Further, plants may deter attackers by reallocating nutrients, such as nitrogen, from vulnerable leaves to tissues that are less accessible, such as the plant roots (Gómez et al. 2010). Many of these mechanical and chemical changes that occur within leaves have potential effects beyond their intended targets because the majority of annual plant production enters the detrital pool where it is consumed by decomposers (Cebrian 1999). Therefore, we aimed to determine how the independent and interactive effects of plant inducible responses via the JA and SA pathways influence decomposer communities in forest soils and small streams.

In terrestrial ecosystems, microbes are the main drivers of litter mass loss, with soil invertebrates also contributing directly through consumption of detritus and indirectly via effects on microbial decomposers (Hättenschwiler et al. 2005). Specifically, the saprotrophic fungi are thought to be the primary decomposers of leaf litter in soils (Lustenhouwer et al. 2020). Given this major role of microbial decomposers in terrestrial systems, rates of litter mass loss may be affected by induction of both the SA and JA pathways because each have been implicated in major shifts in the living plant microbiome (Kniskern et al. 2007). Ultimately, the microbes that remain in a leaf upon senescence are often key in the breakdown of leaf litter (Austin et al. 2014; Peršoh 2013; Voříšková and Baldrian 2013). Studies using the model plant, *Arabidopsis*, have found that experimentally induced upregulation of the SA and JA pathways each alter the abundance and diversity of the leaf microbiome (Kniskern et al. 2007). For example, induction of the SA pathway in *Arabidopsis* increases the relative abundance of many bacterial taxa, including the Flavobacteriaceae, Rhizobiales, Sphingomonadales, Comamonadaceae, and Enterobacteriaceae, which have previously been documented in high relative abundance on decomposing leaves of *A. rubra*, albeit in aquatic ecosystems (Jackrel et al. 2019; Lebeis et al. 2015). Therefore, with the SA pathway having a neutral or beneficial effect on the population abundances of many bacterial taxa associated with decomposition, upregulation of this pathway may in turn have a neutral or beneficial effect on rates of litter mass loss. Furthermore, in terrestrial systems the SA pathway may have a less inhibitory effect on litter mass loss compared to the JA pathway because the former is best known for inhibiting biotrophic pathogens that feed on living plant tissue whereas the latter is known to inhibit necrotrophic pathogens that kill their host plant cells and subsequently decompose dead and dying plant tissue (van Kan 2006).

In aquatic ecosystems, studies have found that leaf mass loss is largely the result of macroinvertebrates, followed by fungi and bacteria (Hieber and Gessner 2002; Marks 2019). However, aquatic macroinvertebrates strongly rely on microbes to condition leaf litter, which improves its nutritional quality (Jonsson and Sponseller 2021). Several studies have shown that intraspecific variation in the anti-herbivory defensive chemistry of trees, particularly genetically controlled concentrations of tannins, is a strong predictor of litter decomposition rates in rivers (LeRoy et al. 2007, 2012) and even the composition and timing of aquatic insect emergence (Compson et al. 2016). Further, aquatic macroinvertebrate detritivores are physiologically similar to terrestrial lepidopteran herbivores in that both have a moderate to highly alkaline gut (Martin et al. 1980, 1981). This suggests that one of the most abundant and diverse classes of anti-herbivore defense compounds that have been found in *Alnus*, the ellagitannins, may have similar detrimental oxidative effects on both terrestrial chewing herbivores and aquatic macroinvertebrate detritivores (Moilanen and Salminen 2008). Furthermore, induction of the JA pathway paired with mock damage from chewing herbivores in *A. rubra* caused shifts in the concentrations of leaf ellagitannins and inhibited litter mass loss in rivers (Jackrel and Wootton 2015). However, there are also notable differences between terrestrial chewing herbivorous insects and aquatic detritivorous insects that may influence how plant defensive compounds affect feeding by these consumers. For example, while aquatic caddisfly and cranefly larvae appear to harbor a resident, core gut microbiome (Jackrel and Broe 2023; Klug and Kotarski 1980), terrestrial lepidopterans have a transient gut microbiome (Hammer et al. 2017). Overall, in both aquatic and terrestrial environments, we test the expectation that induction of the JA pathway will have stronger deleterious effect on litter mass loss than induction of the SA pathway. Furthermore, induction of both the JA and SA pathways simultaneously, can result in additive or non-additive effects on plant performance (Hauser et al. 2013; Zandalinas and Mittler 2022). The interplay between these pathways can be contingent on the order of attackers, identities of the attackers, as well as the host plant identity (Thaler et al. 1999, 2012; van Dijk et al. 2020). So, while plant immune responses can be highly complex in the face of multiple attackers, given that reciprocal antagonism between the JA and SA pathways has been documented in some of the most well-studied model plant systems, we test the expectation that both pathways co-occurring together might yield an intermediate effect.

We evaluated the effects of JA and SA induction through the application of plant hormones on the defensive chemistry and nutrient composition of young *Alnus rubra*, which is the numerically most abundant riparian tree in forests of the Olympic Peninsula of Washington, USA (Furlow 1979; McVean 1953). Application of plant hormones can have differing consequences for plant defensive responses compared to exposure to actual herbivores and pathogens. For example, hormone application lacks the physical damage to plant tissue from chewing or piercing insects, as well as the exposure to chemical compounds found in insect saliva, which can both be important for induction of plant responses (Howe and Jander 2008). Prior induction of mock herbivore defenses in mature *A. rubra* using a mix of methyl jasmonate and mock chewing damage, indicated that these defenses strongly deter terrestrial herbivores and aquatic decomposers, but not terrestrial decomposers (Jackrel and Wootton 2015). Much of this induced defense response to mock herbivory in mature *A. rubra* was a decline in leaf N-content, which is notable considering *Alnus* spp. associate with nitrogen-fixing bacteria (*Frankia alni*) (Benson and Silvester 1993). Here, by using young *A. rubra*, we aim to more fully evaluate the N-based induced defense response in this plant by measuring the nitrogen content of leaves, roots, and N-fixing nodules. For example, we aimed to use measurements of these different plant tissues to evaluate whether the JA induced response results in either the shunting of N from leaves into the roots as a means of resource protection, or alternatively, causes deprioritization of N fixation (Gómez et al. 2010; Sun et al. 2006). Further, we analyzed shifts in *A. rubra* secondary metabolites in response to JA and SA induction by using high-resolution tandem mass-spectrometry. Ultimately, we measure the ecosystem-level implications of these physiological responses to biotic stressors by quantifying effects on litter mass loss in both aquatic and terrestrial ecosystems.

## Methods and Materials

### Study Sites

*Alnus rubra* Bong. is one of the most abundant species of deciduous tree in the riparian zones of the Olympic Peninsula of Washington State, USA. *Alnus* spp. dominate many riparian zones and disturbed forests throughout the Pacific Northwest of North America and across Europe (Furlow 1979; McVean 1953). *Alnus* spp. are able to flourish in disturbed sites in part due to their capacity to use atmospheric nitrogen via symbiotic root associations with the N-fixing bacterium, *Frankia alni* (Benson and Silvester 1993). Previous surveys have shown that *A. rubra* experiences extensive damage from herbivores and pathogens. We found that insects visibly damaged 74% of leaves via skeletonization, leaf miner scars, galls, and rolled and folded leaves from Torticidae moths (Jackrel and Wootton 2015). Their leaves are also frequently infected by pathogenic fungi, including *Gnomonia setacea*, *Gnomoniella tubaeformis*, and *Septoria alni* (Sieber et al. 1991).

We used a naturally occurring group of young *A. rubra* trees growing in an early successional forest patch of the Merrill & Ring Pysht Tree Farm (48.098 N, 124.128 W). The South Fork of the Pysht River runs through this tree farm. This 3rd -order river has a riparian zone dominated by *A. rubra,* bigleaf maple (*Acer macrophyllum* Pursh.) and western hemlock (*Tsuga heterophylla* Raf. Sarg.). Understory vegetation throughout much of the tree farm is a mix of salal (*Gautheria shallon* Pursh.), salmonberry (*Rubus spectabilis* Pursh.), vine maple (*Acer circinatum* Pursh,), and sword fern (*Polystichum munitum* Kaulf.). Additional location and environmental characteristics of the Pysht River are reported elsewhere (Jackrel and Wootton 2014; Wootton 2012).

To evaluate the immediate implications of JA and SA-induced responses on litter mass loss, and due to the abundance and importance of greenfall in this ecosystem, we chose to focus on green rather than senescent leaves. Surveys of leaf litter fall into other rivers in the region during summer have found that *A. rubra* comprised 95–98% of litter fall, of which 60–87% was green in color rather than brown senescent litter (Jackrel and Wootton 2014). Temperate rainforests receive as much as 20% of their annual leaf flux as green rather than senescent litter (Campbell and Fuchshuber 1994). Although still a small proportion of total annual leaf fall, green litter is more nutrient dense, with higher concentrations of nitrogen, phosphorus and potassium, and has been implicated in seasonally variable aquatic nutrient cycling and growth of aquatic macroinvertebrates (McArthur et al. 1986; Risley and Crossley Jr 1988). For example, nutrient-dense greenfall has resulted in fourfold greater concentrations of phosphorus inputs in summer (McArthur et al. 1986) and the availability of green leaf litter has been implicated in faster growth among aquatic macroinvertebrates (Kochi and Kagaya 2005; Stout et al. 1985).

### Experimental Design

We implemented a 2 × 2 experimental design to test the independent and interactive effects of JA and SA pathway induction on *A. rubra* physiology and subsequently leaf litter mass loss. We completed the experiments in two consecutive rounds, with the first 20 individuals receiving their assigned treatment in June 2013 and the second 20 individuals receiving their assigned treatment in July 2013. Treatments consisted of an individual tree receiving one of four chemical treatments: either JA pathway induction via methyl jasmonate, SA pathway induction via salicylic acid, both pathways via a mixture of methyl jasmonate and salicylic acid, or a water-based solution as a control. We then followed a similar protocol to what we had used previously for mature *A. rubra *(Jackrel and Wootton 2015). Specifically, we collected four leaves per tree prior to the chemical treatment as baseline measures of leaf traits. We then immediately brushed every third leaf on the tree with 50 µl of their assigned solution, each of which contained 10% ethanol and 0.125% Triton-X, as well as either 100 mM methyl jasmonate, 100 mM salicylic acid, 100 mM of both compounds, or neither compound as a control. Leaves brushed with these treatments were also marked with a dot of white paint. We repeated this procedure three times over a 4-day period in June or July, with the same marked leaves treated each time. By the end of the treatment, one-third of all leaves on the tree were brushed with their assigned solution three times (Jackrel and Wootton 2015). By repeating this procedure, we intended to mimic a sustained threat to the plant rather than a single pulse of damage, which has often failed to induce strong defense responses in plants (Mithöfer et al. 2005). In Round 1, we collected 30 leaves from each tree on June 14, 2013 for leaf trait measurements, leaf pack assembly, and deployment in aquatic and terrestrial ecosystems. We also collected additional leaves and samples of roots and N-fixing nodules when uprooting the trees on June 25, 2013. Although removing 30 leaves from each tree may have caused defoliation stress, this pressure would have been uniform across treatment groups. For Round 2, we collected 30 leaves from each tree on July 9, 2013 for leaf trait measurements, leaf pack assembly and leaf pack deployment in aquatic and terrestrial ecosystems, and then immediately uprooted trees on the same day to sample roots and root nodules. Average trunk diameter of the 40 trees was 1.03 ± 0.04 cm at 1.0 m above ground, which likely corresponds to saplings under 2 years of age (Ericsson and Rytter 1998). Tree diameters were similar across treatment groups (*F*_3,30_ = 1.49, LMER *P* > 0.05) and experimental rounds (*F*_1,30_ = 0.86, LMER *P* > 0.05). Immediately prior to applying our plant hormone treatments, trees were also similar in leaf chemistry across treatment groups (all linear mixed effects models *P* > 0.05 for % N – *F*_3,31_ = 0.30, C: N – *F*_3,31_ = 0.25, δ^13^C – *F*_3,31_ = 1.92 and δ^15^N – *F*_3,31_ = 1.27 across treatment groups), but did vary by round (i.e. month) in %N, C:N and δ^15^N (ANOVAs: *P* < 0.05 for Round, % N – *F*_1,31_ = 5.78, C: N – *F*_3,31_ = 4.67, δ^15^N – *F*_3,31_ = 7.14).

When picking leaves for trait measurements and leaf pack assembly, we chose leaves that showed little or no visible damage from herbivores and pathogens. We only used leaves that were not brushed with our chemical treatment because we aimed to test the systemic effects of the treatment, as well as to avoid any effects that the remaining chemical residue may have on decomposers. We used 24 of the leaves collected per tree for immediate assembly into two replicate leaf packs from each of the 40 individual trees for deployment in aquatic and terrestrial ecosystems. As our aim was to understand the effects of phenotypic variation in green rather than senescent litter, we chose to deploy fresh green leaves without oven- or air-drying. To minimize leaf desiccation during transport, leaves were sealed in plastic zip-top bags immediately after detachment from the tree. We also avoided a pre-drying step to minimize damage to heat-sensitive plant secondary metabolites. For each leaf pack, 12 leaves were placed in a bag that we constructed from 4.75 mm delta mesh knotless polyester netting (Memphis Net & Twine), which permits colonization by most soil and stream macroinvertebrates.

All leaf packs were deployed within 24-hours of leaf collection from *A. rubra* individuals. To measure litter mass loss in the terrestrial system, initial leaf mass was recorded and sets of leaf packs were deployed using steel reinforcing bars within the same forested area as the study trees. We covered packs with 1.25 cm of debris from the immediate area. After 38 and 37 days of incubation for Round 1 and 2 respectively, we washed the leaves in water to dislodge soil invertebrates and silt. We then oven-dried leaves and weighed to the nearest 0.01 g. To measure litter mass loss in the aquatic system, initial leaf mass was recorded and leaf packs were strung with cable ties onto a steel reinforcing bar that was laid on the streambed perpendicular to the water flow in the South Fork Pysht River (48.167°N, 124.157°W). Leaf packs were deployed for 17 or 15 days for Rounds 1 and 2, respectively. We chose this shorter duration because, based on our prior work in these rivers, alder leaves are often nearing extensive decomposition by 20 days, as indicated by a skeletonized leaf with minimal remaining structure.

As described further under statistical analysis, our prior lab studies demonstrated that final leaf weights should be prepared differently for terrestrial versus aquatic environments (Jackrel and Wootton 2014). Therefore, to measure final mass of leaves submerged in the aquatic environment, we gently washed leaves with water to dislodge aquatic macroinvertebrates and sediment, blotted leaves dry with paper towels, and weighed to the nearest 0.01 g. Leaf pack location was randomized within each of the aquatic and soil deployment sites to minimize any systematic differences in the biotic and abiotic conditions affecting each treatment group and control for any mass gain or loss due to sedimentation.

#### Plant Trait Measures

For nutrient and isotopic analyses, we followed our previously used methods for *A. rubra* (Jackrel and Wootton 2015). In brief, we used four leaves per tree collected immediately before and after experimental treatments. We oven dried leaves at 40 °C immediately after collection, ground them into a fine powder, and archived leaf powder in the dark at ~ 20 °C until further analysis. Archived materials were then used in 2020 for nutrient and isotopic analyses. Specifically, we packed 3 mg of power into 3.5 × 5 mm tin capsules. Measures of %N, %C, C:N, δ^15^N, and δ^13^C were determined using a PDZ Europa ANCA-GSL elemental analyzer interfaced to a PDZ Europa 20–20 isotopic ratio mass spectrometer at the University of California Davis Stable Isotope Facility.

Using the same leaf material collected for isotopic measurements, we also measured secondary metabolite composition of four fresh green leaves that were collected from each of the 40 trees immediately before and after our plant hormone treatments using the mass spectrometry methods described in Jackrel and Morton (2018). In brief, we used the leaf powder prepared as described above that had been oven dried at 40 °C to minimize damage to heat sensitive secondary metabolites. We extracted 100 mg of leaf powder in 10 mL 70% methanol and auto injected samples through an Agilent Zorbax SB-C18 2.1 × 150 mm, 3.5 μm column on a Q-Exactive quadrupole orbitrap mass spectrometer (Thermo Fisher Scientific) through the University of California San Diego Microbiome and Metabolomics Core. Samples were eluted with 0.1% formic acid in water (A) and 0.1% formic acid in acetonitrile (B) using the following separation gradient: 5 min of 100% A at a rate of 0.2 mL/min, followed by a gradient from 100 to 60% A (0 to 40% B) over 20 min at a rate of 0.1 mL/min. Flow was then held for 5 min at 60% A (40% B) at a rate of 0.3 mL/min. Then a gradient from 60 to 0% A (40 to 100% B) was carried out over 15 min at 0.3 mL/min, and then held for 10 min at 0% A (100% B) at 0.3 mL/min. Compounds were characterized using retention times and fragmentation patterns of chromatograms using the Global Natural Products Social Molecular Networking (GNPS) analysis platform, a comparison against our previously developed library of *A. rubra* metabolites as described in Jackrel and Morton (2018), and a review of the literature (Wang et al. 2016).

#### Statistics

To determine which of our measured plant traits best separated treatment groups, we used discriminant function analysis (linear discriminant analysis [LDA]), which is a supervised method that aims to maximize between-group separation, using the lda function in the R package MASS. To minimize the effects of experimental round on plant traits and instead focus on treatment effects, we converted all predictor variables to standard scores (i.e. z-scores) for each experimental round. As many of the secondary metabolites identified in our study are building blocks of one another, or closely related isomers, multicollinearity among traits should be expected. To reduce multicollinearity for a more robust LDA, we focused our analyses on the more abundant compounds in our dataset that could both be quantified more accurately and have a higher likelihood of biological effects. Specifically, we retained variables that both had a mean total ion account above 100, and those that did not strongly co-vary (i.e., >0.70) with other abundant compounds: including 38 plant secondary metabolites described in Table S2 for our first and third LDA, described below, and 41 plant secondary metabolites described in Table S3 for our second LDA. Additionally, two of our 80 mass spectrometry samples were omitted from our analyses due to poor data quality. To correct for the unbalanced design that this created with unequal samples per treatment groups, we dropped samples from the LDA analysis as needed. Specifically, as trees were randomly assigned treatment groups within blocks of four trees that were geographic neighbors, when there was an incomplete block of four trees, additional samples within that same block were dropped to generate a balanced design.

We determined the significance of discriminant functions using the Wilk’s lambda statistic with a Chi square distribution, where smaller values indicate a smaller proportion of the total variance among discriminant scores that is not explained by differences among groups (Ripley 2007). We carried out three discriminant function analyses. To compare the relative roles of leaf secondary metabolites versus plant nutrient traits on separation of treatment groups, our first analysis incorporated all leaf secondary metabolite, leaf nutrient, root nutrient and nodule nutrient traits (*n* = 48 traits in total). As root and nodule measurements required destructive sampling, this analysis only evaluated traits post treatment. For our second analysis, we aimed to control for standing variation in plant chemistry that occurred among trees before applying our hormone treatments. Therefore, this analysis focused on paired measurements of leaf secondary metabolites from the same individual tree taken both before and after treatment. Finally, we did a third analysis to generate discriminant scores for use in our stepwise linear mixed effects models to determine which traits best predicted litter mass loss. This LDA included only leaf secondary metabolite traits post treatment because nutrient traits were used as independent variables in model selection. For each LDA analysis, we then determined all pairwise significant differences between groups using post hoc Tukey’s HSD tests where groups that significantly differ are shown using 50% confidence ellipses of the discriminant scores.

To test which plant traits best predicted rates of litter mass loss in rivers and soils, we used linear and linear-mixed effects models using the lme function in the R package nlme. Traits tested in these models include nutrient traits of leaves, roots and nodules, including C:N, δ^15^N, δ^13^C, and leaf secondary metabolite traits, summarized using discriminant scores. To select best fitting models, we used forward automated model selection using the stepAIC function in the R package MASS and compared AIC scores. For best fitting models, we report the marginal R^2^ value to describe the proportion of variance explained by the fixed factors (Nakagawa and Schielzeth 2013). Due to the unusual mechanism of litter mass loss observed in the second round of aquatic leaf packs, as described further in the results, we ran a linear model on only the first round of litter mass loss. Due to high multicollinearity between root and nodule nutrient traits in this dataset for the first round, we dropped nodule traits to reduce variance inflation factors below 3.4 (µ_VIF_ = 1.67). This was preferable to dropping root traits, because this approach retained relatively high variance inflation factors of up to 8.1 (µ_VIF_ = 3.28). For soils, we completed a linear mixed effect model with experimental round as a random effect term. To remain consistent with our models for aquatic litter mass loss, we also dropped nodule traits for our models for litter mass loss in soils but dropping these traits had no effect on final model selection.

To ensure accurate measurements of leaf mass loss, we needed to consider moisture levels of our initial and final leaf masses. This is especially important for leaf mass loss studies in rivers, which necessarily require that leaves are incubated in different conditions (i.e., submerged in water) than those in which leaves were initially collected (i.e., exposed to air). To do this, we used data from our previously performed laboratory-based experiment described by Jackrel and Wootton (2014) to determine the most accurate method for measuring change in grams of leaf mass loss rather than water content. The majority of leaf pack studies use dried senescent leaf litter from autumnal leaf fall and can therefore employ air or oven drying to bring leaf litter to a constant initial mass prior to deployment (Hieber M, Gessner MO 2002; LeRoy et al. 2020; Marks et al. 2009). However, as we aimed to understand leaf mass loss of green fall, pre-drying leaf litter was not desirable because this practice fundamentally changes the properties of fresh green leaves in unnatural ways prior to deployment. To find alternative methods that were both usable for fresh green leaves and applicable to aquatic and terrestrial leaf pack studies, we previously collected 12 leaves from 19 individuals of *A. rubra.* Leaf masses were recorded in groups of six leaves per tree at three timepoints: (1) fresh green leaves, (2) submerged in buckets of tap water for 17 days and then blotted dry with paper towels, and (3) oven-dried to a constant mass. The second set of six leaves per tree were oven-dried before and after water submergence to estimate mass loss due to leaching, handling and measurement-error. We found that the strongest correlation occurred between pre- and post- oven dried leaves (*R*^*2*^ = 0.991). This indicated minimal mass loss caused by leaching, handling and/or measurement error. However, we found nearly as strong of a correlation between fresh leaf mass and the mass of leaves blotted dry with paper towel (R² = 0.983). In contrast, we found an appreciably weaker correlation between fresh leaf mass and oven dried leaf mass (R² = 0.875). Therefore, in the current study we used these relationships drawn from our laboratory-based work to back-calculate fresh mass of leaves following incubation in the rivers: fresh mass = 0.941(blotted-dry mass) − 0.00337 (*R*^*2*^ = 0.98). In contrast to litter deployed in rivers, we have observed that litter deployed in soil systems is often unevenly saturated with moisture due to variation in sun exposure, soil texture, and other microclimatic differences. Therefore, we determined oven-drying to be the most accurate means for measuring mass loss in a soil system and used the following equation: fresh mass = 2.53(oven dried-mass) + 0.275 (*R*^*2*^ = 0.79) (Jackrel and Wootton 2014).

## Results

### Plant Hormone and Seasonally Induced Shifts in *A. rubra* Composition

**Fig. 1:**
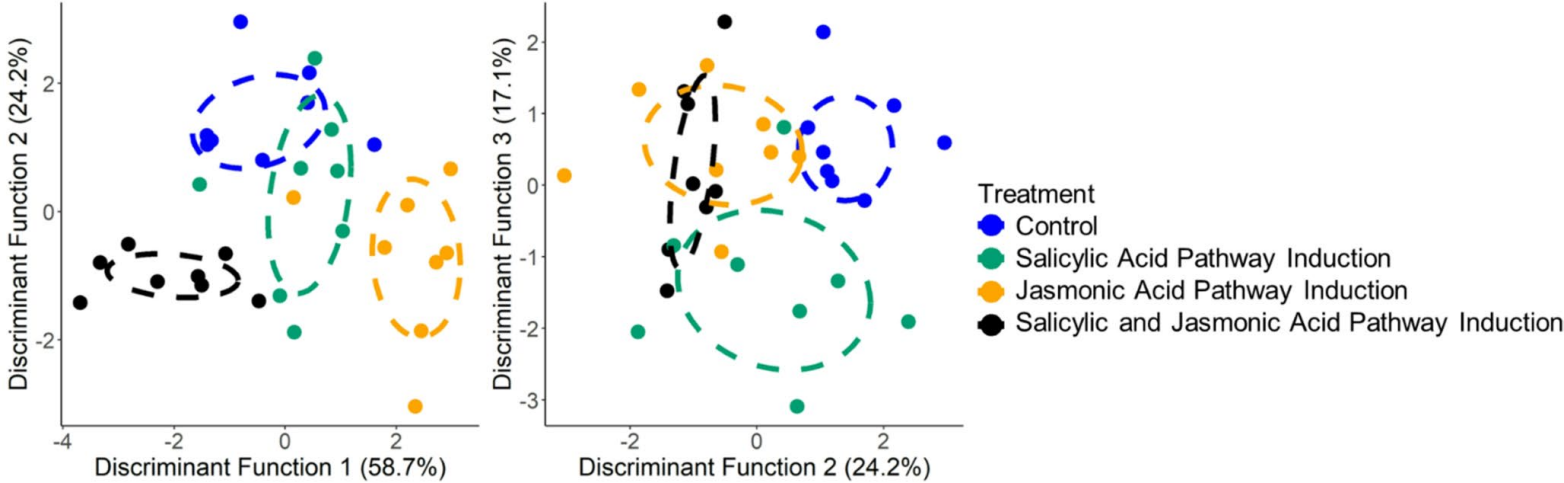
Separation in the secondary metabolite composition of leaves and the nutrient stoichiometry of leaves, roots and root nodules of young *Alnus rubra* trees treated with the plant hormones methyl jasmonate and/or salicylic acid to mimic biotic stress from herbivores and pathogens. Significant separation among groups shown using (**A**) the DF1 and 2 (DF1: *Wilk’s* λ = 0.271, *F* = 25.1, *P* < 0.001; DF2: *Wilk’s* λ = 0.475, *F* = 10.3, *P* < 0.001), and (**B**) DF2 and 3 (DF3: *Wilk’s* λ = 0.561, *F* = 7.3, *P* < 0.001). See Table S2 for variable coefficients, showing which traits most strongly weight each discriminant function

Young *A. rubra* diverged significantly in secondary metabolite and stoichiometric composition following treatment with the plant hormones methyl jasmonate and/or salicylic acid to induce the JA and SA pathways, respectively. In particular, trees experiencing JA induction either with or without SA induction tended to diverge from the control group (Fig. 1, DF1: *Wilk’s λ* = 0.271, *F* = 25.1, *P* < 0.001; DF2: *Wilk’s λ* = 0.475, *F* = 10.3, *P* < 0.001; DF3: *Wilk’s λ* = 0.561, *F* = 7.3, *P* < 0.001). The JA treatment group diverged along DF1, which corresponded with increased concentrations of the larger ellagitannins tellimagrandin II and tellimagrandin β, and reduced concentrations of the ellagitannin 2x HHDP-glucose (Fig. 1, Table S2). While trees in the JA treatment group differed markedly from trees experiencing combined induction of both pathways along DF1, these two groups converged on DF2 and DF3, with both groups tending to have elevated concentrations of a relatively large ellagitannin tentatively identified as rhoipteleanin H (Jiang et al. 1999).

Although treatment groups were best distinguished by discriminant analysis based on divergence among plant secondary metabolites, there were also significant differences among treatment groups in plant stoichiometry (Table S2; see pairwise differences among treatments in Fig. S3). Specifically, leaves from trees in the JA treatment group tended to contain a lower %N and elevated C: N relative to the other treatments. This shift in nitrogen was apparent among leaves collected four days after applying the treatments in rounds 1 and 2, when leaf litter packs were assembled (JA treatment: mean = 3.00%*N* ± 0.072 *SE* and 15.9 C: *N* ± 0.37 *SE* vs. other treatments: 3.12%*N* ± 0.047 *SE* and 15.2 C: *N* ± 0.23 *SE*), and was especially notable among leaves collected when the young trees were uprooted sixteen days after the start of treatments in Round 1 (JA treatment in Round 1: mean = 2.67%*N* ± 0.14 *SE* and 17.8 C: *N* ± 1.04 *SE* vs. other treatments in Round 1: 3.29%*N* ± 0.08 *SE* and 14.5 C: *N* ± 0.38 *SE*; Fig S3, linear mixed effects models for both rounds: %N – *P* = 0.012, *F*_3,30_ = 4.17, and C: N – *P* = 0.012, *F*_3,30_ = 4.24). Root nodules of trees in the JA treatment group also contained lower %N and elevated C: N relative to other treatments (JA treatment pooled across both rounds: 2.05%*N* ± 0.15 SE, 21.4 C: *N* ± 1.1 *SE* vs. other treatments in both rounds: 2.31%*N* ± 0.08 *SE*, 19.1 C: *N* ± 0.50 *SE*; linear mixed effects models for both rounds: %N – *P* = 0.14, *F*_3,30_ = 1.81; C: N – *P* = 0.069, *F*_3,30_ = 2.77). Additionally, leaves and root nodules from trees in the JA treatment group tended to have relatively low δ^13^C (Fig S3; linear mixed effects models for both rounds: Leaves *P* = 0.034, *F*_3,30_ = 2.63; Nodules *P* = 0.018, *F*_3,30_ = 3.20).

**Fig. 2:**
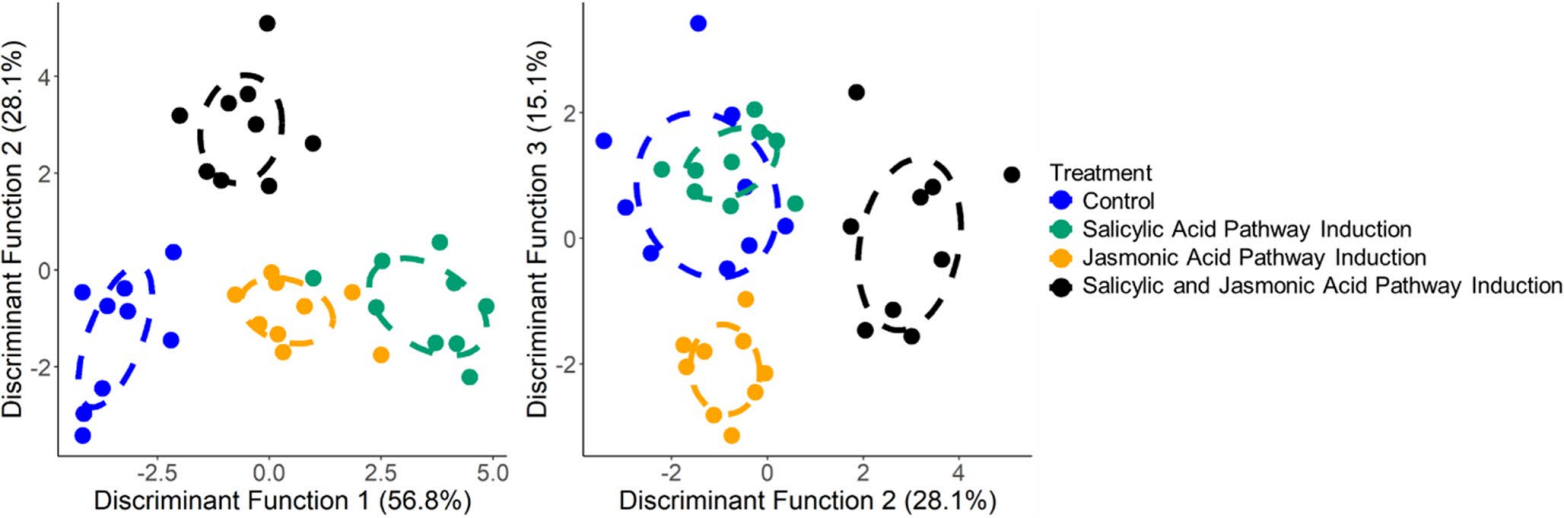
Trait shifts among the leaves of *Alnus rubra* trees after treatment with the plant hormones methyl jasmonate and/or salicylic acid to induce the jasmonic acid and/or salicylic acid defensive pathways in response to biotic stress from herbivores and pathogens. Percentage shift in secondary metabolites is reported for each tree using paired data collected from leaves from each individual tree before and after the assigned treatment. Significant separation among groups shown using (**A**) the DF1 and 2 (DF1: *Wilk’s* λ = 0.129, *F* = 72.28, *P* < 0.001; DF2: *Wilk’s* λ = 0.230, *F* = 35.8, *P* < 0.001), and (**B**) DF2 and 3 (DF3: *Wilk’s* λ = 0.357, *F* = 19.2, *P* < 0.001). See Table S3 for variable coefficients, showing which traits most strongly weight each discriminant function

To better understand the physiological shifts in leaf secondary metabolite composition, for which it was possible to have paired measurements from each tree before and after treatment, we ran a second discriminant function analysis. We found significant separation among treatments in leaf secondary metabolite composition (Fig. 2, DF1: *Wilk’s λ* = 0.129, *F* = 72.3, *P* < 0.001; DF2: *Wilk’s λ* = 0.230, *F* = 35.8, *P* < 0.001; DF3: *Wilk’s λ* = 0.357, *F* = 19.2, *P* < 0.001). In particular, the SA treatment group diverged most strongly along DF1 compared to the control group, which corresponded with the greatest changes for the SA group in the concentrations of five diarylheptanoids (including increases in alnuside A, hirsutanonol, and aceroside VIII, and declines in oregonin and oregonoyl A). The JA and SA combined treatment and the JA alone treatment both were similar along DF1. However, these two groups diverged along DF2 where the combined treatment diverged from all other treatment groups based on a greater decline in the ellagitannin precursor tetra-galloyl-glucose, alnuside B glycoside, and an unknown diarylheptanoid (#52), as well as a less steep decline in another unknown diarylheptanoid (#44) compared to the other treatment groups. In contrast, the JA treatment group diverged on DF3 from all other treatment groups based on an increase in tetra-galloyl-glucose, which as the biosynthetic precursor to tellimagrandin β, is in line with the high final concentration of tellimagrandin β found in the JA treatment group, as shown in Fig. 1.

**Fig. 3:**
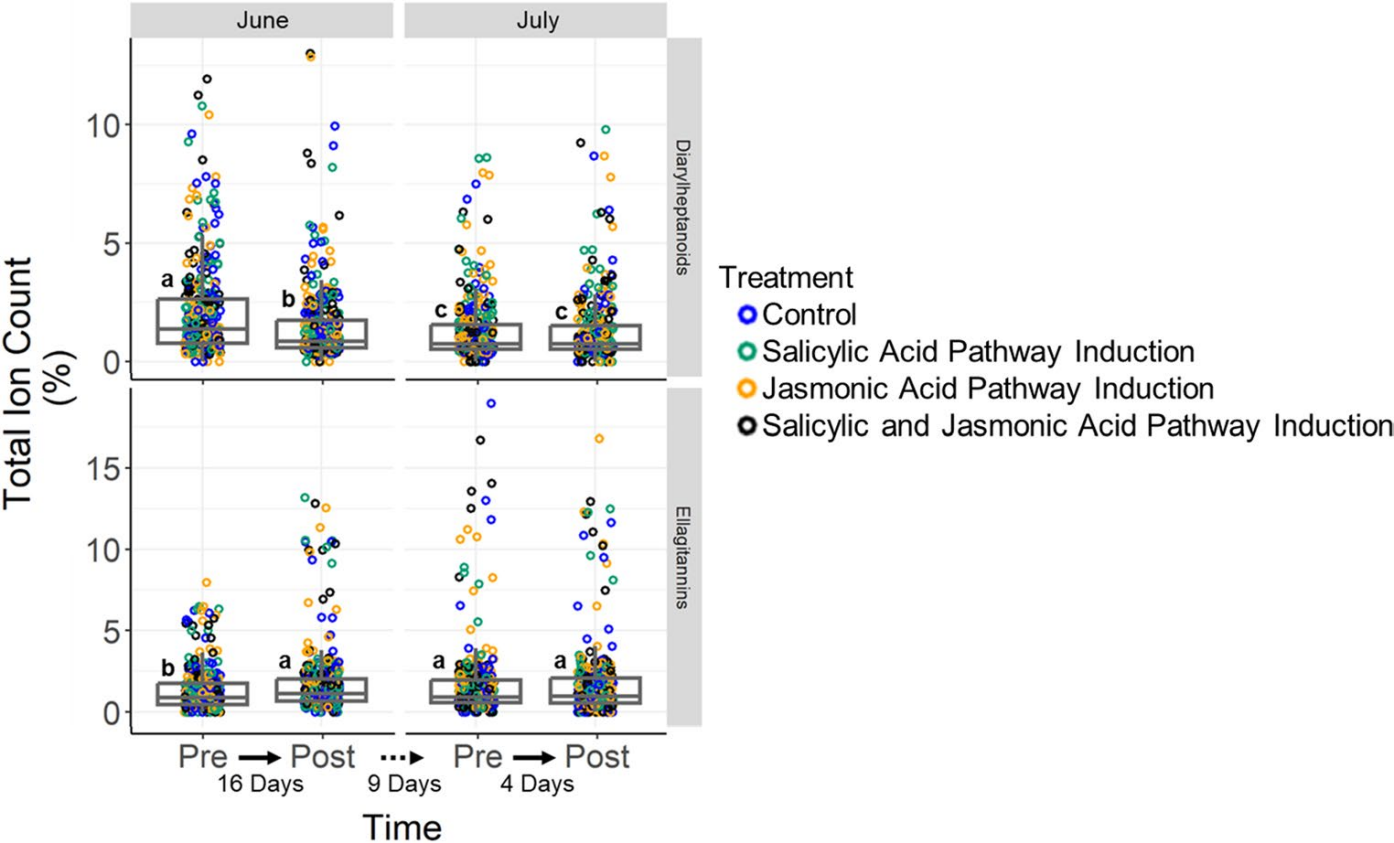
Seasonal changes in the relative concentrations of two families of plant secondary metabolites in the green leaves of *Alnus rubra* trees show opposing trends. The cytotoxic diarylheptanoids decline significantly from mid to late June, whereas the dosage dependent ellagitannins increase over this same time period. Labelling of the x-axis indicates whether each measurement was taken before (pre) or after (post) trees were treated with plant hormones. Linear mixed effects models were run for each chemical family and each experimental round with time point as a fixed effect and molecular compound nested within tree ID as a random effect (June – diarylheptanoids *P* < 0.0001, *F*_1,655_ = 42.58; July diarylheptanoids *P* > 0.05, *F*_1,587_ = 0.011; June – ellagitannins *P* < 0.0001, *F*_1,733_ = 33.45; July – ellagitannins *P* > 0.05, *F*_1,657_ = 0.032). To illustrate seasonal changes across months, linear mixed effects models were also run with time point (i.e. four in total across both months) as a fixed effect and molecular compound as a random effects term with lettering to indicate post-hoc differences

In addition to the treatment-specific shifts in leaf secondary metabolites, we also found that the two major families of these plant secondary metabolites showed opposing trends over the duration of the growing season. Specifically, diarylheptanoids showed a gradual decline in relative concentration over the duration of the growing season (Fig. 3; LMER: *P* < 0.0001, *F*_3,1272_ = 65.57). In contrast, ellagitannins increased sharply in relative concentration in mid-to-late June and then remained at these elevated concentrations through July (Fig. 3; LMER: *P* < 0.0001, *F*_3,1422_ = 23.03).

### Ecosystem Effects of *A. rubra* Composition

**Fig. 4:**
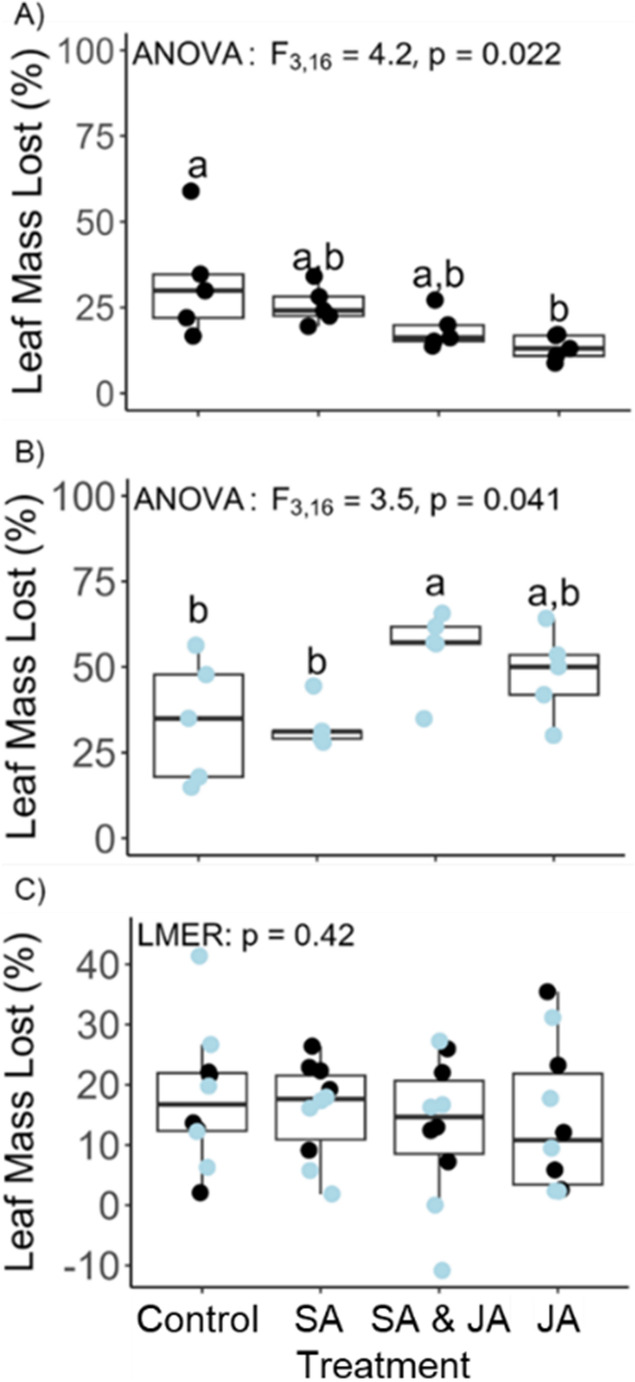
Leaf litter mass losses (%) from *Alnus rubra* trees growing on the Olympic Peninsula, Washington, USA that were treated with plant hormones to evaluate the effects of induced stress responses to herbivores and pathogens on litter breakdown. Trees were treated with methyl jasmonate and/or salicylic acid to induce the jasmonic acid (JA) and salicylic acid (SA) defense pathways, respectively. Aquatic litter mass loss measured in the Pysht River, Washington during (**A**) June, at which time typical litter breakdown via skeletonization was observed, and (**B**) July, when an abnormally high number of *Dicosmoecus* caddisflies were present and removed large sections of leaf tissue rather than skeletonization. (**C**) Terrestrial litter mass loss was also measured in both the June (black) and July (blue) rounds in forest soil nearby the *A. rubra* trees used in the study. A linear mixed effects model was used for terrestrial litter mass loss with deployment site nested within experimental round as the random effect. Treatment groups sharing the same letter do not significantly differ according to Tukey’s post-hoc tests

Patterns of litter mass loss in an aquatic ecosystem were markedly different depending on experimental round, likely due to an unusual steep increase in the abundance of *Dicosmoecus* caddisflies between the June and July 2013 deployment dates. The higher density of caddisflies in July corresponded with a visible shift in the mechanism of leaf breakdown. In all other field studies that we have carried out at these sites over the past decade, *A. rubra* leaf mass loss resulted from a gradual thinning of layers that are sheared off presumably by microbes and small invertebrates, leaving a delicately skeletonized pattern of all the major and minor leaf veins. However, in July during the presence of an exceptionally high abundance of *Dicosmoecus* caddisflies (the cause of which was unknown), the leaves had whole bites removed that were approximately 6 mm in diameter, leaving behind no skeletonized leaf (S.L. Jackrel personal observation). This shift in mechanism was also accompanied by an overall increase in litter mass loss. In Round 1, average leaf mass lost across treatment groups was 21.1% ± 2.3 *SE* after 17 days of incubation, while in Round 2, leaf mass lost averaged 42.9% ± 3.1 *SE* after only 15 days of incubation (ANOVA: *F*_1,42_ = 29.0, *P* < 0.0001). During Round 1, patterns of leaf mass loss matched our a priori expectation with leaves from trees given the SA treatment breaking down more than leaves from trees given the JA treatment, while leaves from trees treated with both plant hormones lost mass at an intermediate level (Fig. 4A, ANOVA *F*_3,16_ = 4.2, *P* = 0.022). During Round 2, patterns of litter mass loss varied by treatment groups, but did not match our a priori expectation (Fig. 4B, ANOVA *F*_3,16_ = 3.5, *P* = 0.041). Terrestrial leaf mass loss did not differ significantly by treatment group, however patterns of litter mass loss matched results seen in Round 1 for aquatic ecosystems with greatest mass loss among the SA treatment group, lowest mass loss among the JA treatment group and intermediate mass loss among the combined plant hormone group (Fig. 4C, LMER *P* = 0.42).

**Fig. 5:**
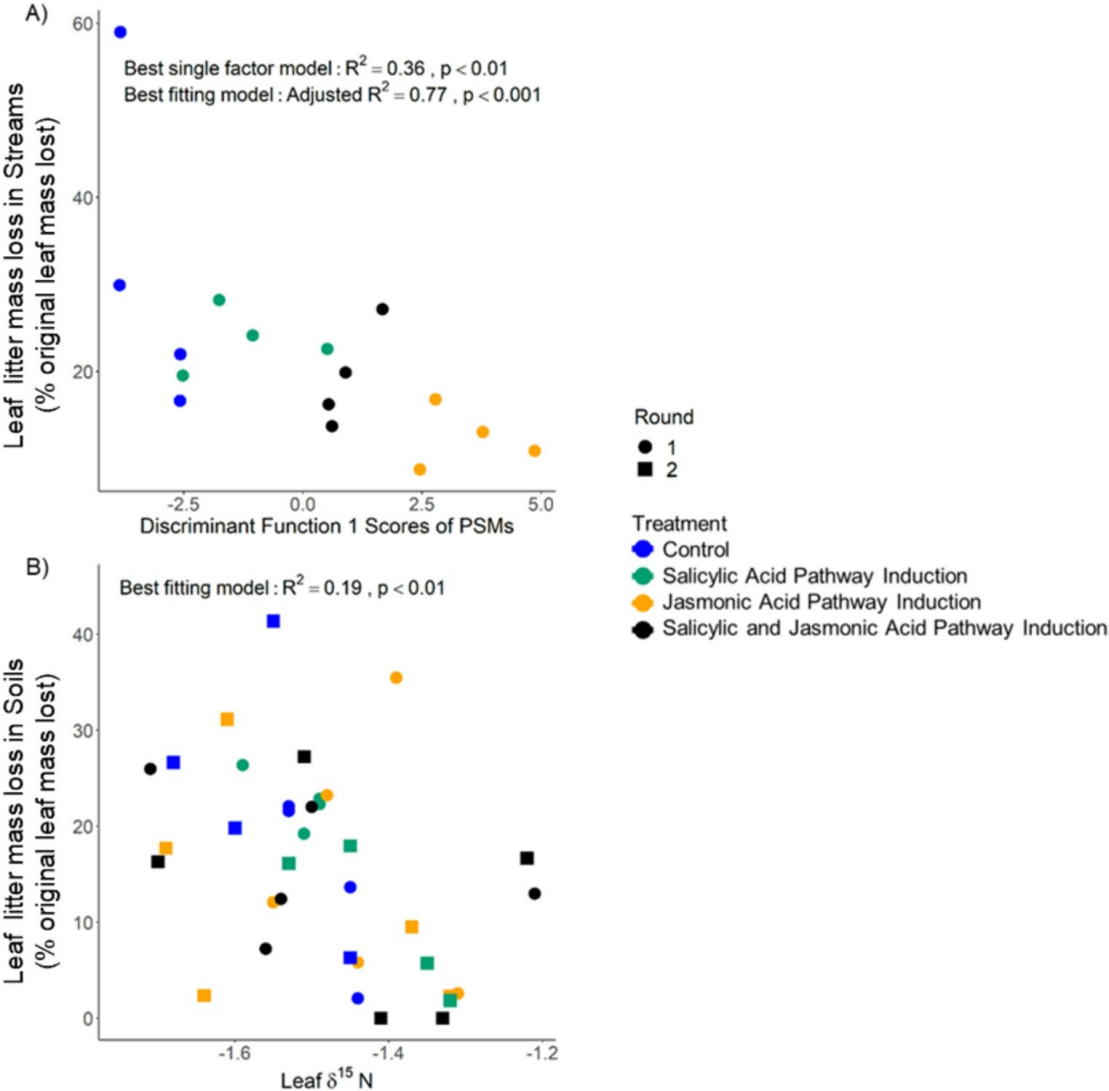
Leaf litter mass losses (%) from *Alnus rubra* trees in (**A**) rivers were best predicted by variation in plant secondary metabolites (PSM), as summarized by the discriminant function reported in Fig. S1, while litter mass losses in (**B**) soils were best predicted by leaf δ^15^N. Aquatic litter mass loss was best predicted by a multivariate linear model that also included root δ^15^N, discriminant function 3 from Fig S1, leaf δ^13^C, and root C: N. The best fitting linear mixed-effects model for terrestrial litter mass loss, which included experimental round as a random effect, was a single variate model with leaf δ^15^N

To determine which traits of trees best predicted litter mass loss in aquatic and terrestrial environments, we used stepwise regression on all stoichiometric and secondary metabolite measurements taken post treatment of leaves, root tissue and root nodules. Secondary metabolite composition post treatments were summarized as discriminant function scores for each of the three axes shown in Fig. S1 (DF1: *Wilk’s λ* = 0.144, *F* = 63.5, *P* < 0.001; DF2: *Wilk’s λ* = 0.156, *F* = 57.6, *P* < 0.001; DF3: *Wilk’s λ* = 0.295, *F* = 25.6, *P* < 0.001). Mass loss in aquatic ecosystems was best predicted by a model of the first discriminant function of secondary metabolites as well as root δ^15^N, the third discriminant function of secondary metabolites, leaf δ^13^C, and root C: N (Fig. 5A, Linear Model: adjusted *R*^*2*^ = 0.78, *P* < 0.001). Litter mass loss in an aquatic ecosystem could also be significantly predicted by a single variate model of the first discriminant function alone, which was most heavily weighted by an unknown metabolite, alnuside B glycoside, an unknown ellagitannin, aceroside VIII, and tellimagrandin II (Fig. 5A, Fig S1, adjusted *R*^*2*^ = 0.36, *P* < 0.01). As we aimed to understand how tree physiology regulated typical patterns of leaf mass loss in this system, we only used mass loss values from the aquatic ecosystem from Round 1. Litter mass loss in the terrestrial ecosystem was best predicted by leaf δ^15^N (Fig. 5B, *R*^*2*^ = 0.13, *P* = 0.051).

## Discussion

Our results demonstrate that terrestrial plant responses to simulated biotic stress can have implications for the composition and eventual breakdown of leaf litter in aquatic ecosystems. Specifically, we find that induction of the JA and SA pathways trigger distinct defensive responses among plants that reshape the composition of leaf secondary metabolite and nutrient traits, with cascading effects on leaf litter mass loss in river ecosystems, and to a lesser extend in soil ecosystems . Although breakdown rates in these terrestrial versus aquatic ecosystems could be best predicted by different plant traits, ultimately, we found that patterns of litter mass loss in both ecosystems were similar. In both systems, the greatest breakdown rates were observed for litter originating from SA-induced trees whereas the least breakdown was observed for litter originating from JA-induced trees. Further, simultaneous induction of both the JA and SA pathways resulted in a plant defensive response that was distinct from the plant responses to either pathway alone and resulted in values of litter mass loss that were intermediate between those values from plants experiencing induction of either pathway in isolation.

Leaf litter breakdown results from the activities of diverse consortia of bacteria, fungi and macroinvertebrates. Therefore, different aspects of plant defenses against terrestrial pathogens and/or invertebrate herbivores could have varied implications on different members of the detritivore community. Consistent with our prior work with mature *A. rubra*, we found that a substantial component of the induced defensive response among young trees involved a decline in leaf nitrogen (Jackrel and Wootton 2015). Further, young *A. rubra* induced via the SA pathway retained significantly higher leaf %N, while trees receiving the combined plant hormone treatment were intermediate in %N content. While prior work with mature *A. rubra* mimicked chewing herbivory by combining both methyl jasmonate treatment with mechanical leaf damage, our current results demonstrate that this decline in nitrogen can be triggered solely from hormonal signaling without mechanical leaf damage. Further, by using young trees, we were able to compare %N across plant tissues. We found that %N of root nodules among treatments matched the ordering observed in leaves (i.e. lowest in the JA treatment group and highest in the SA treatment group). In contrast, %N in root tissue showed the opposite pattern, with highest %N among the JA treatment group and lowest %N among the SA treatment group. Studies with the *A. rubra* congener, *Alnus incana* (L.) Moench, found that the highest concentrations of N in root nodules tended to occur in response to N-depleted soils and corresponded with high levels of nitrogenase, which is the enzyme responsible for N_2_ fixation (Gentili and Huss-Danell 2003). While additional studies are necessary to determine the physiological mechanism underlying the shift in nitrogen we have observed in *A. rubra*, our results suggest that JA-induced trees may have shunted nitrogen from the leaf tissue to the root tissue, which tended to be highest among JA-induced trees, and the resulting excess of N reallocated to belowground tissues may have deprioritized N fixation, as suggested by low %N in root nodules and less negative leaf δ^15^N.

The N-based defensive response to mock chewing herbivory via JA induction was more detectable in the first experimental round. This may have been due to the relatively longer period of time between treatments and final measurements, during which measurable physiological shifts could accumulate (i.e. 16 days post treatment versus 4 days post treatment). It is also conceivable that *A. rubra* stress responses vary seasonally, perhaps due to lower average leaf %N early in the growing season, as seen in Fig. S2 and in other *Alnus* spp. (Dawson and Funk 1981), which could make the benefits of defending a scarcer resource outweigh the energetic expense of upregulating defense (Harper 1989; Meyer and Montgomery 1987). Our prior documentation of the shift in leaf N due to JA induction in mature *A. rubra* took place early in the growing season, similar to the first experimental round in the present study. Another possibility is that over the duration of a growing season, trees accumulate variable levels of exposure to natural ecological stressors, causing greater standing variation in traits that could make detection of trends against a noisy background more challenging. However, because our pre-treatment measurements of trees indicated greater standing trait variation during the first experimental round, especially for %N and C: N, it appears that trends should have been detectable had they occurred rapidly during the shorter period of time tested in this second round (i.e. 4 days).

Beyond the N-based induced defensive response to mock chewing herbivory, we also found that *A. rubra* employs its diverse phytochemistry to mount highly tuned responses to, not only the type, but also to the combination of stressors targeting the plant. Notably, induction of the JA pathway seems to largely result in the restructuring of ellagitannin composition. This association between the JA pathway and ellagitannin composition was expected given recent advancements in our mechanistic understanding of how these dosage-dependent chemical deterrents could decrease chewing herbivory due to strong oxidation activity (Salminen and Karonen 2011). Strong oxidants reduce nutritional quality by increasing metabolic costs within the alkaline guts of many chewing insect herbivores (Salminen and Karonen 2011). Other recent studies have identified shifts in ellagitannin concentration in a range of plant species in response to changes in insect chewing herbivore pressure (Agrawal et al. 2012; Anstett et al. 2019; Jackrel and Morton 2018). However, we have also shown that particularly when resources become more limited, *A. rubra* will predominantly employ diarylheptanoids in defensive responses against mock chewing herbivory pressure (Jackrel and Morton 2018). In contrast to this heavier reliance on ellagitannins as part of the JA pathway, induction of the SA pathway appeared to trigger more substantial changes in the concentrations of diarylheptanoids, a class of potent cytotoxins that have been targets for drug development (Alberti et al. 2018; Novaković et al. 2014). While the ecology of diarylheptanoids remains poorly understood and new molecules within this class are still frequently being discovered, recent studies have found that most diarylheptanoids that have been isolated from *Alnus* spp. have potent antibacterial and antifungal properties (Ilic-Tomic et al. 2017; Novaković et al. 2015). This restructuring of diarylheptanoid composition in response to SA pathway induction may therefore be effective at reducing the population sizes of a range of plant bacterial and fungal pathogens. However, despite the seemingly broad effectiveness of these compounds against multiple bacterial phyla, including the proteobacteria and actinobacteria, we did not find evidence that induction of the SA pathway results in effective inhibition of leaf litter mass loss in either aquatic or terrestrial environments. Considering that SA induction has been implicated in inhibition of biotrophic but not necrotrophic plant pathogens, this induced defensive response may have limited effects on microbial decomposers. Future work should investigate whether SA-induced compositional shifts among the diarylheptanoids have any cascading effects on the aquatic bacterial and fungal communities that decompose leaf litter in river and soil systems.

In addition to the differential regulation of these families of secondary metabolites in response to plant hormone treatment, our results further suggested that these two families may serve differing ecological roles across the growing season. The consistent decline in concentration across the family of diarylheptanoids over the duration of the growing season suggests that these toxins serve as a more important defense system early in the growing season, when resources may be limited and the value of young plant tissue is higher. For example, our measures of leaf nitrogen immediately before applying hormone treatments indicated that plants were more N-limited early in the growing season. This heavier reliance on diarylheptanoids in resource limited environments aligns with our past work that found mature *A. rubra* responded to mock chewing herbivory stress by regulating diarylheptanoids when nutrients were limited, and instead via regulation of ellagitannins when nutrients were supplied in excess by fertilizing with phosphorus (Jackrel and Morton 2018). More generally, juvenile plants and younger leaf tissues tend to be defended by greater concentrations of plant secondary defenses (Karban and Myers 1989; Lambdon and Hassall 2005). One explanation for this elevated level of defense is that young leaves present early in the growing season on trees with indeterminant growth, such as *Alnus* and *Betula* spp., may be more intensely defended due to their higher expected lifetime value to the parent plant from prolonged potential for future photosynthesis and energy allocation towards additional growth (Harper 1989; Meyer and Montgomery 1987). Further, chemical defenses may be greater among younger leaves to compensate for lower levels of physical defenses, such as leaf toughness (Coley 1983; Meyer and Montgomery 1987). In contrast, the increase in a quantitative defense like ellagitannins later in the growing season may be advantageous when baseline resources, particularly nitrogen levels, are more available. The increase we observed in ellagitannins over the growing season is consistent with a high-resolution study of seasonal variation in ellagitannin concentrations of *Betula pubescens* Ehrh. that found that the most abundant ellagitannins in these trees increased substantially over the growing season (Salminen et al. 2001). Broadly, our results align with expectations from plant apparency theory that long-lived plants with excess resources favor the use of dose-dependent deterrents whereas short-lived plants and those with more limited resources favor more potent toxins (Feeny 1976; Novaković et al. 2014).

We also found that the effects of litter compositional quality on rates of mass loss can be context dependent. Aquatic decomposers were more affected by JA-induced plant responses than SA-induced plant responses during our first round of the study, which was when we observed the typical mechanism of litter breakdown in rivers of the Olympic Peninsula of Washington of gradual thinning and leaf skeletonization. However, we found conflicting results in our second round when *Dicosmoecus* caddisflies were exceptionally abundant. This context dependency suggests that under conditions of lower population density (and presumably lower competition for limited resources), decomposers have significant preferences regarding resource quality. However, during periods of caddisfly over-population, which we are inferring based on our observations of typical densities in this system, resource scarcity reduces the importance of resource quality. Further, in soil systems we observed the same ordering of litter mass loss by treatment across both rounds of the experiment as observed in aquatic systems during the first round of the experiment (i.e. typical litter breakdown). However, soil decomposer communities appeared less affected overall by either stressor, which is in line with our prior findings that JA-induced responses in mature *A. rubra* had weaker effects on decomposition rates in soils compared to rivers (Jackrel and Wootton 2015).

Overall, there is substantial evidence that within-species biodiversity can have wide ranging implications across trophic levels and ecosystem boundaries (Crutsinger et al. 2006; Madritch and Hunter 2002; Schweitzer et al. 2005; Whitham et al. 2006). Deciphering whether the traits driving these effects are the consequence of fixed genetic differences versus inducible phenotypic variation is particularly important for long-lived species as it can shed light on the stability of ecosystem function over time. Decades of research using the common garden experimental forest of cottonwoods in Utah, USA has demonstrated the potential for cascading effects of tree genotype, including genotypically-controlled induced responses, on adjacent river systems (LeRoy et al. 2007, 2012; Marks et al. 2009). Such controlled experiments are invaluable for quantifying the proportional contributions of genotype versus plasticity on ecosystem functions. In contrast, experiments using naturally recruited populations of plants can clarify whether these effects are detectable within a system of greater standing variation reflective of the natural environment. Using *A. rubra*, we have shown in our prior work that constitutive defensive chemistry traits can have measurable outcomes for not only aquatic ecosystem function and community composition of bacterial decomposers, but also have broader effects on fitness among macroinvertebrate consumers in natural systems (Jackrel and Broe 2023; Jackrel et al. 2016, 2019). We now demonstrate that simulated biotic stresses on *A. rubra* mimicked through the use of plant hormones to induce the JA and SA pathways rapidly reshape the composition of leaf nutrient and defensive chemistry traits and that these induced shifts subsequently alter rates of litter mass loss. It is therefore likely that plant responses to biotic stressors within natural ecosystems could have broader implications across ecosystem boundaries. Understanding the relative importance of genetically hardwired versus inducible trait variation in driving rates of ecosystem function is an important next step that could improve the ability to predict the stability and resilience of these functions across space and time.

## Supplementary Information

Below is the link to the electronic supplementary material.

Supplementary Material 1

## Data Availability

All mass spectrometry data is publicly available on MassIVE under project number MSV000087609. All additional data and metadata is available at: 10.5281/zenodo.16877969.
