# Supplementary material for: Cascading Effects of Plant Hormone-induced Trait Shifts in Alnus rubra on Aquatic and Terrestrial Ecosystem Function
Source: J Chem Ecol. 2025 Sep 13;51(5):92. doi: 10.1007/s10886-025-01644-9 (PMC12431895; doi:10.1007/s10886-025-01644-9)

CASCADING EFFECTS OF PLANT HORMONE-INDUCED TRAIT SHIFTS IN *Alnus rubra* ON AQUATIC AND TERRESTRIAL ECOSYSTEM FUNCTION

TARYN Y. BROE,^#^ ALEXIA FABIANI,^#^ MIRTE C.M. KUIJPERS^#^ AND SARA L. JACKREL*

*School of Biological Sciences, Department of Ecology, Behavior & Evolution*

*University of California San Diego, 9500 Gilman Drive #0116*

*La Jolla, CA, 92093-0116*

^#^ Co-first authors

* Corresponding author: sjackrel@ucsd.edu

**SUPPLEMENT**

**Table S1** Peak number, mean total ion count (TIC), retention time (RT), [M − H]- of parent molecule and diagnostic ions (including exact mass and percentage) of 61 plant secondary metabolites found in a population of 40 young *Alnus rubra* trees in NW Washington, USA. For compounds that could be tentatively identified, we note the chemical and common name, general category of plant secondary metabolite, and method of characterization. For parent molecules with an identification, we report the MS2 fragments of the parent ion. When a parent molecule could not be identified, we report the MS1s. For peak # 40, identification was based on what we interpreted as the [M+Cl]- rather than the [M − H]- (see Dinic et al. 2016).


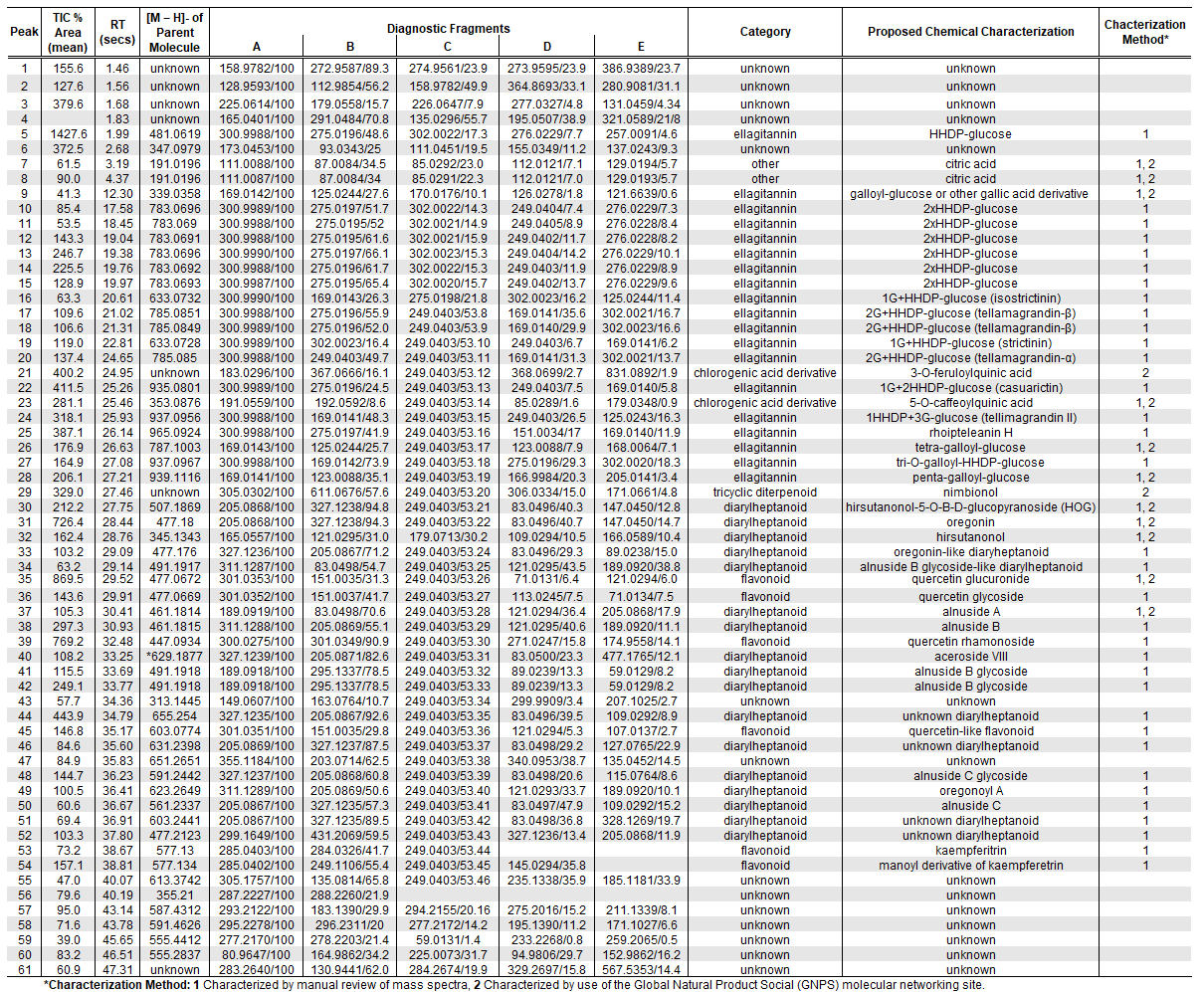


**Table S2** Coefficients for discriminant functions illustrated in Fig. 1. To focus this analysis on compounds found at higher relative abundance and reduce multicollinearity, we excluded traits with a mean TIC below 100 (as described in Table S1). To reduce multicollinearity, we excluded additional traits with high collinearity, R^2^ > 0.70, specifically: # 30 hirsutanonol-5-O-B-D-glucopyranoside due to collinearity with #14 2x HHDP-glucose, #31 oregonin and #42 alnuside B glycoside; # 37 alnuside A due to collinearity with #5 HHDP-glucose; # 41 alnuside B glycoside due to collinearity with # 44 unknown diarylheptanoid; and Root δ^13^C due to collinearity with Nodule δ^13^C.


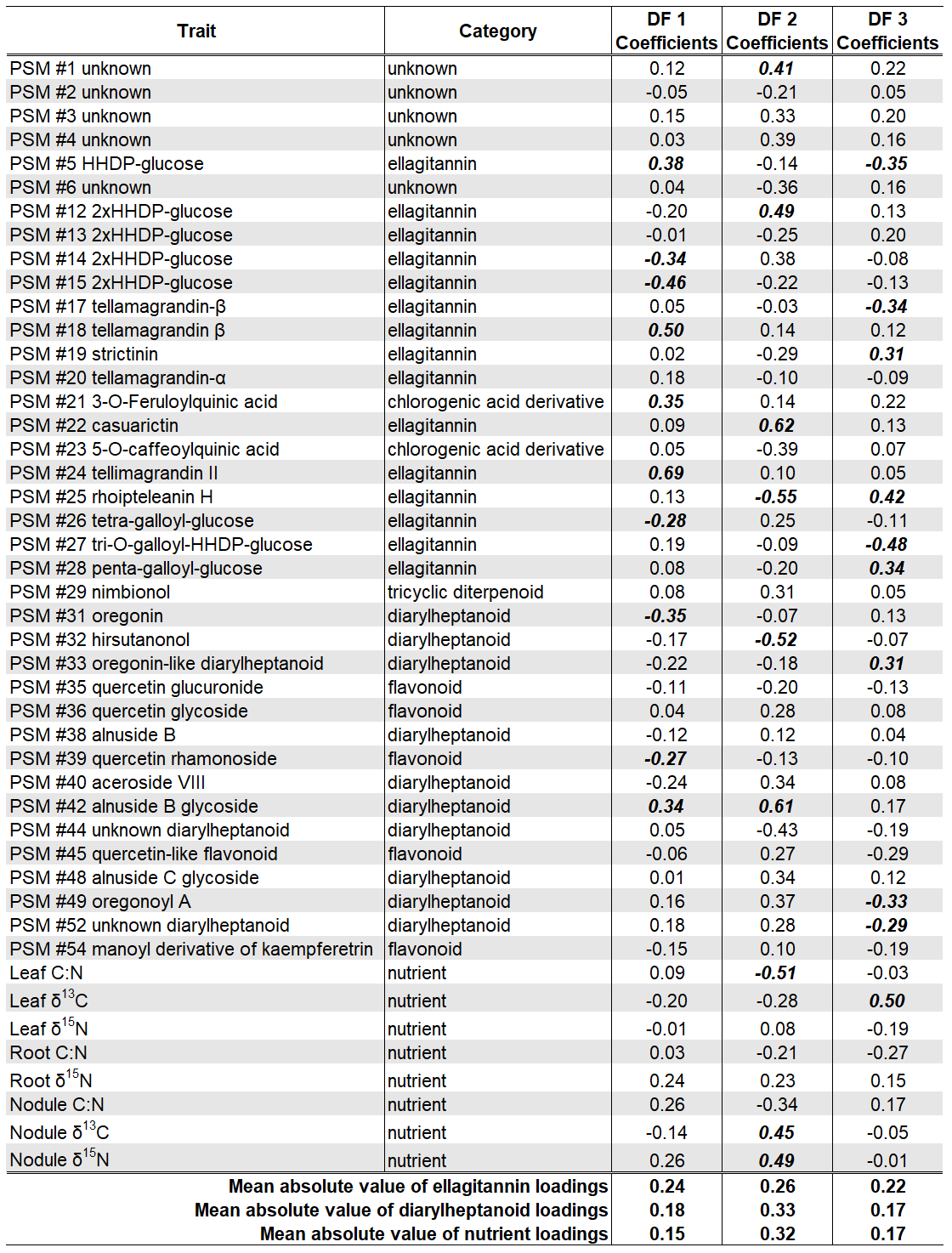


**Table S3** Coefficients for discriminant functions illustrated in Fig. 2. To focus this analysis on compounds found at higher relative abundance and reduce multicollinearity, we excluded traits with a mean TIC below 100 (as described in Table S1). None of the remaining traits showed high collinearity (> 0.70) and so no additional exclusions were necessary.

**
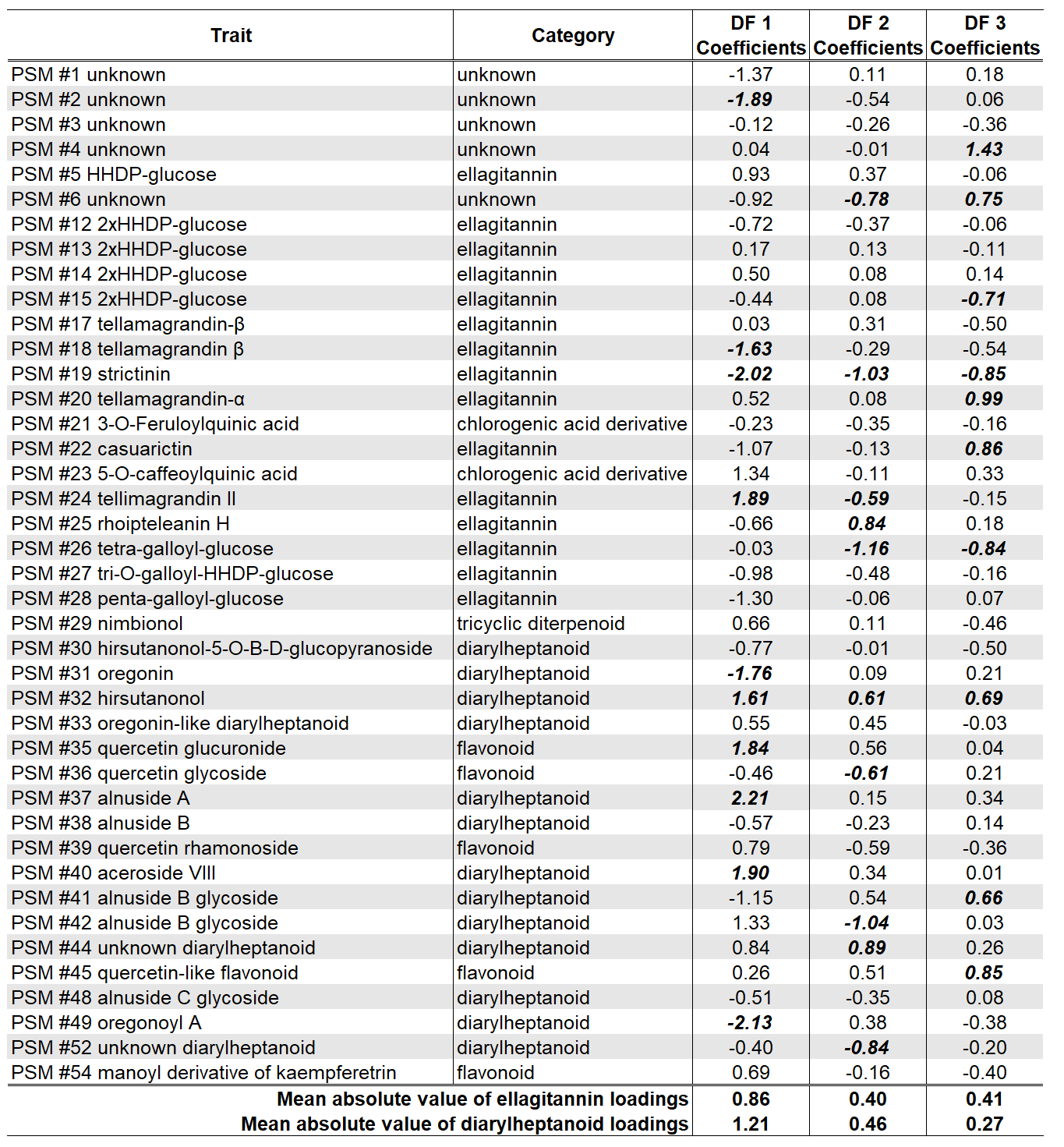
**

**Fig. S1** Divergence in leaf secondary metabolite composition of *Alnus rubra* trees after treatment with the plant hormones methyl jasmonate and/or salicylic acid to induce the jasmonic acid and/or salicylic acid defensive pathways in response to biotic stressors, including herbivores and pathogens. Significant separation among groups shown using A) the DF1 and 2 (DF1: *Wilk’s λ* = 0.151, *F* = 52.4, *P* < 0.001; DF2: *Wilk’s λ* = 0.250, *F* = 28.1, *P* < 0.001), and B) DF2 and 3 (DF3: *Wilk’s λ* = 0.587, *F* = 6.58, *P* = 0.002). All pairwise differences between groups are significant (*P* < 0.01) on at least one DF axis. See Table S4 for variable coefficients, showing which traits most strongly weight each discriminant function. These discriminant functions were used as summary variables in a stepwise regression model to determine which leaf traits best predict rates of litter decomposition in aquatic and terrestrial environments.


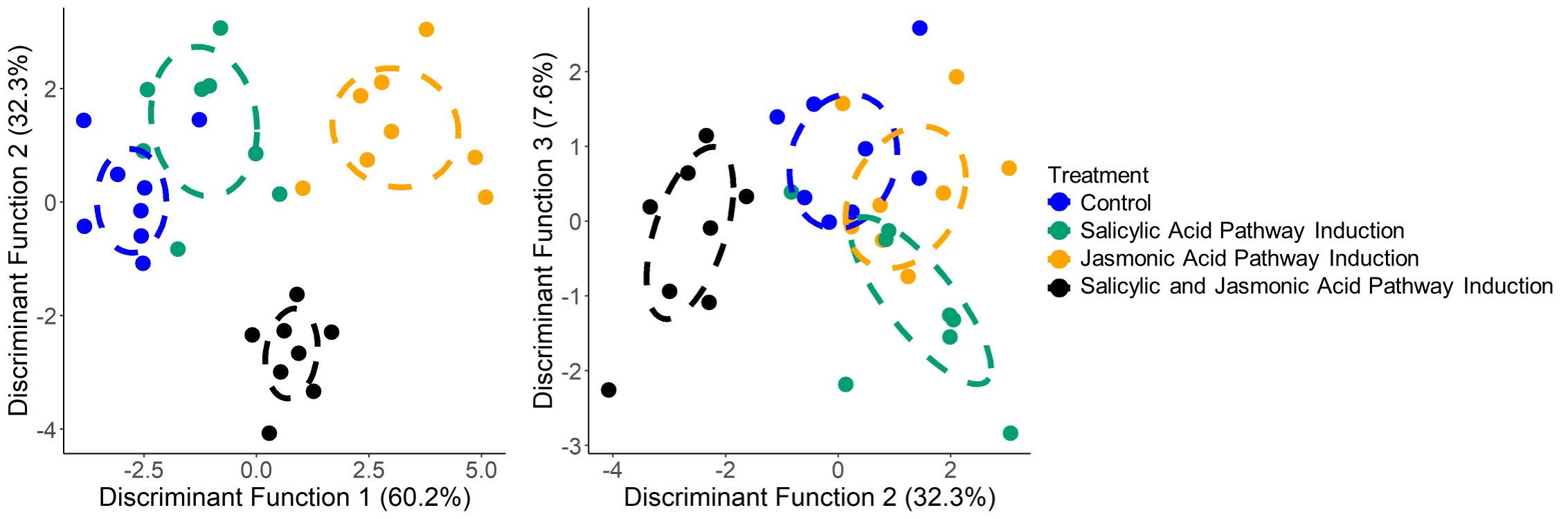


**Table S4** Coeifficents for discriminant functions illustrated in Fig S1. To focus this analysis on compounds found at higher relative abundance and reduce multicollinarity, we excluded traits with a mean TIC below 100 (as described in Table S1). To reduce multicollinarity, we excluded additional traits with high collinearity, R^2^ > 0.70, specificially: # 30 hirsutanonol-5-O-B-D-glucopyranoside due to collinearity with #14 2x HHDP-glucose, #31 oregonin and #42 alnuside B glycoside; # 37 alnuside A due to collinearity with #5 HHDP-glucose; and # 41 alnuside B glycoside due to collinearity with # 44 unknown diarylheptanoid.

**
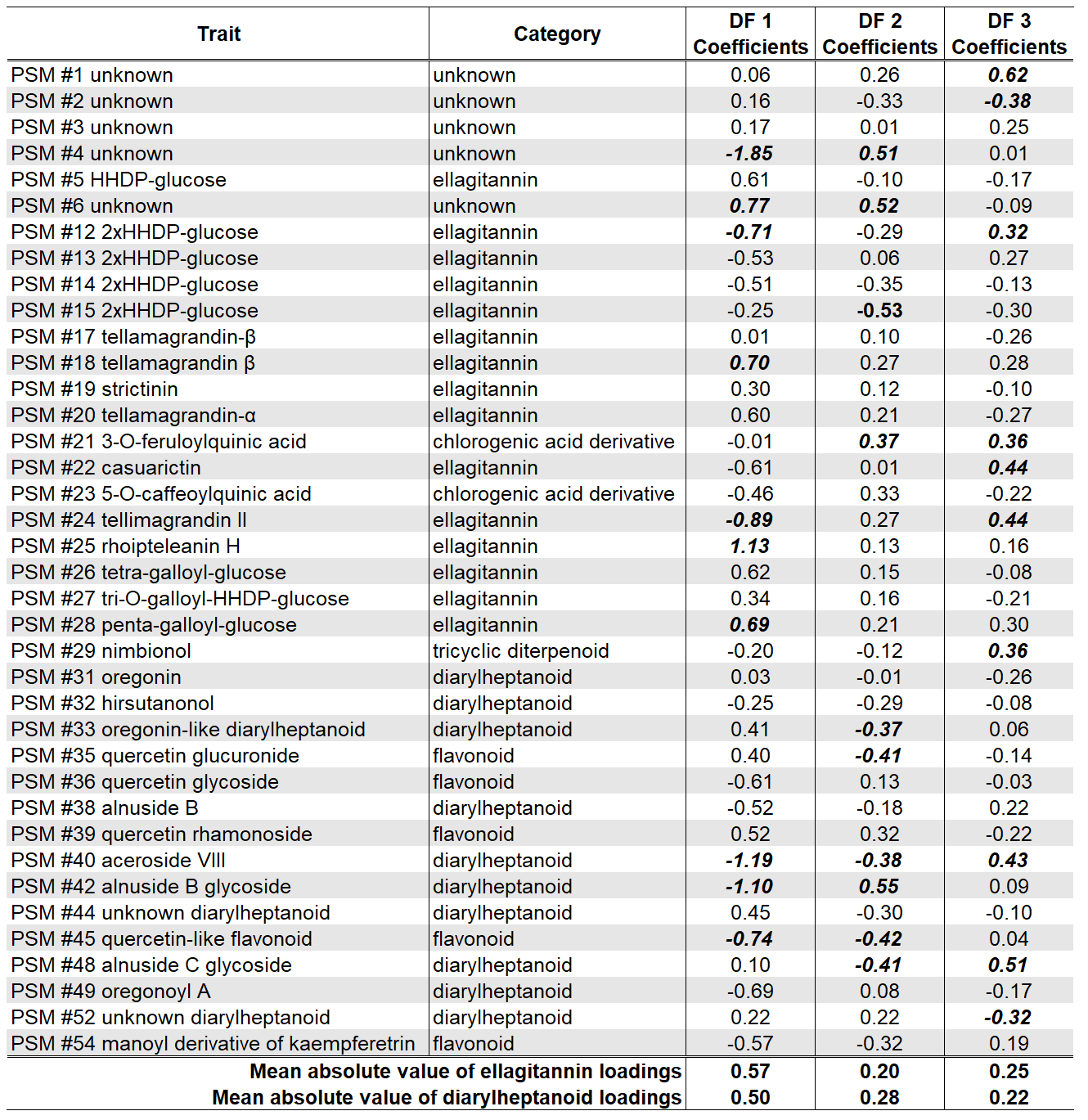
**

**Fig. S2** Immediately prior to treating *A. rubra* trees with plant hormone treatments including methyl jasmonate to induce the jasmonic acid (JA) pathway and/or salicylic acid to induce the salicylic acid (SA) pathway, leaves were collected to measure **A)** %N, **B)** C:N, **C)** δ^15^N and **D)** δ^13^C. Linear mixed effects models with assigned treatment as a fixed effect and experimental round as a random effect were run for each metric. Samples from the first round of the study shown in black and samples from the second round of the study shown in blue.

**
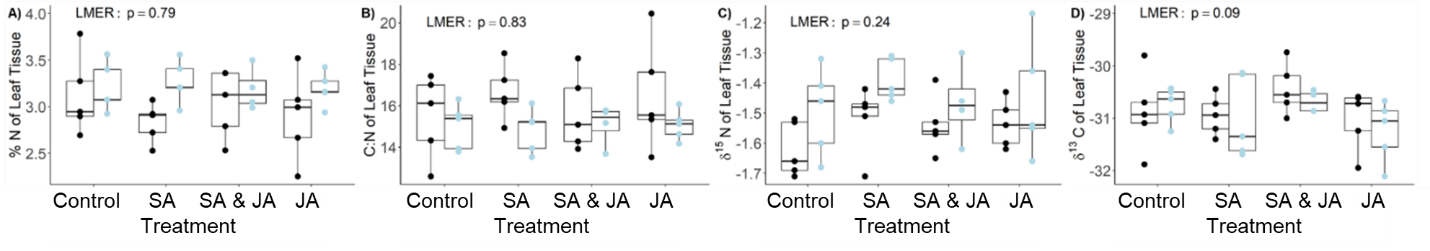
**

**Fig. S3** After treating young *A. rubra* trees with methyl jasmonate to induce the jasmonic acid (JA) pathway, salicylic acid to induce the salicylic acid (SA) pathway, both, or a water control containing neither, leaves were collected and trees were uprooted to sample root tissue and root nodules harboring N-fixing microbial symbionts. Percentage nitrogen was measured in **A)** leaf tissue, **B)** root tissue, and **C)** root nodules. C:N, δ^15^N and δ^13^C were also measured in leaves, roots and nodules as shown in **D) – L)**. Linear mixed effects models with treatment as a fixed effect and experimental round as a random effect were run for each metric and tissue type. Treatment groups sharing the same letter do not significantly differ according to Tukey’s post-hoc tests at *P* < 0.10 level. Samples from the first round of the study shown in black and samples from the second round of the study shown in blue.


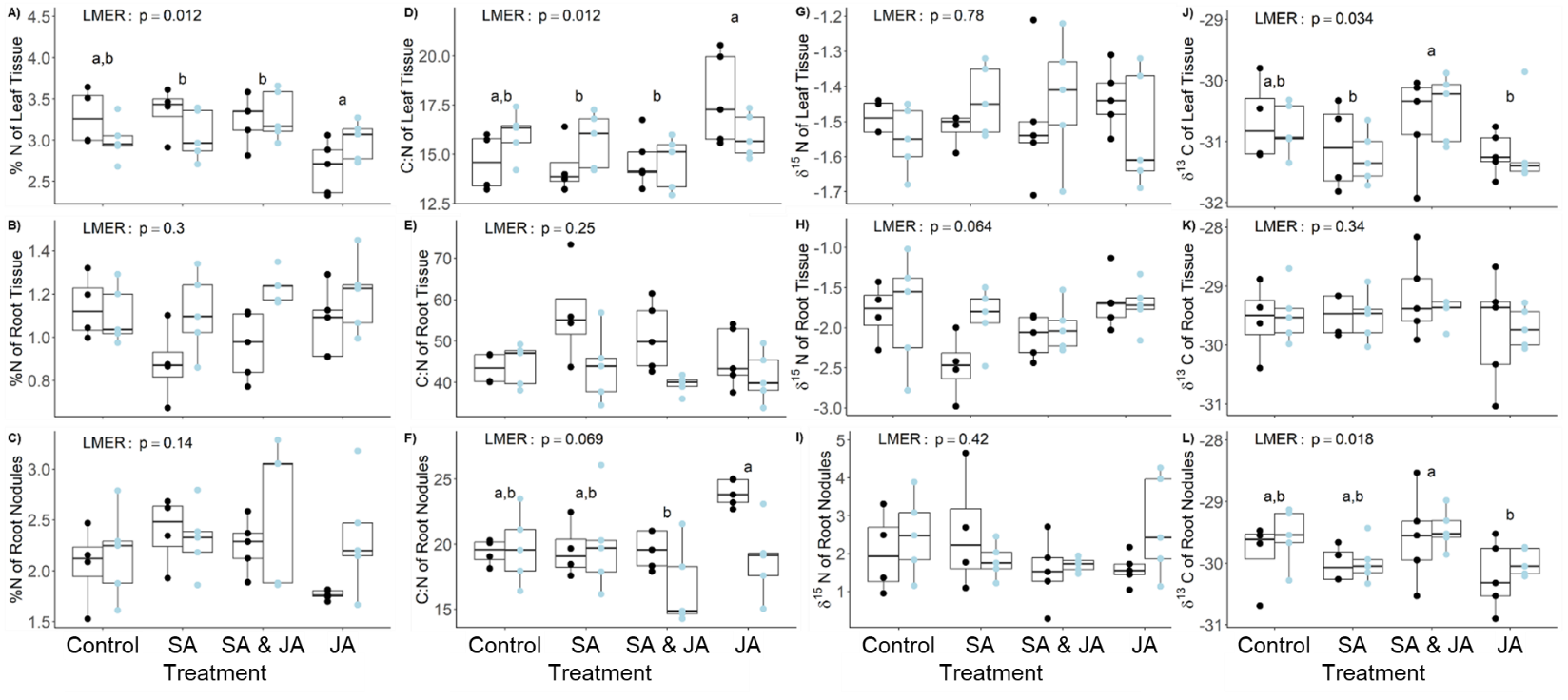

Supplement: Supplementary file 1 — Supplementary Material 1 [file 10886_2025_1644_MOESM1_ESM.docx]
